# Epiregulin enhances odontoblastic differentiation of dental pulp stem cells via activating MAPK signalling pathway

**DOI:** 10.1111/cpr.12680

**Published:** 2019-08-27

**Authors:** Dixin Cui, Jiani Xiao, Yachuan Zhou, Xin Zhou, Ying Liu, Yiran Peng, Yi Yu, Hongyu Li, Xuedong Zhou, Quan Yuan, Mian Wan, Liwei Zheng

**Affiliations:** ^1^ State Key Laboratory of Oral Diseases, National Clinical Research Center for Oral Diseases, Department of Pediatric Dentistry West China Hospital of Stomatology, Sichuan University Chengdu Sichuan China; ^2^ State Key Laboratory of Oral Diseases, National Clinical Research Center for Oral Diseases, Department of Cariology and Endodontics West China Hospital of Stomatology, Sichuan University Chengdu Sichuan China; ^3^ State Key Laboratory of Oral Diseases, National Clinical Research Center for Oral Diseases, Department of Implantology West China Hospital of Stomatology, Sichuan University Chengdu Sichuan China

**Keywords:** dental pulp stem cells, epiregulin, mitogen‐activated protein kinase signalling pathway, odontoblastic differentiation

## Abstract

**Objectives:**

The odontoblastic differentiation of dental pulp stem cells (DPSCs) contributes to tertiary dentin formation. Our previous study indicated that epiregulin (EREG) enhanced odontogenesis potential of dental pulp. Here, we explored the effects of EREG during DPSC odontoblastic differentiation.

**Methods:**

The changes in EREG were detected during tertiary dentin formation. DPSCs were treated with recombinant human EREG (rhEREG), EREG receptor inhibitor gefitinib and short hairpin RNAs. The odontoblastic differentiation was assessed with ALP staining, ALP activity assay, alizarin red S staining and real‐time RT‐PCR of *DSPP, OCN, RUNX2* and *OSX*. Western blot was conducted to examine the levels of p38 mitogen‐activated protein kinase (p38 MAPK), c‐Jun N‐terminal kinase (JNK) and extracellular signal‐regulated kinase 1/2 (Erk1/2). The expression of *EREG* and odontoblastic differentiation‐related markers was investigated in human dental pulp from teeth with deep caries and healthy teeth.

**Results:**

Epiregulin was upregulated during tertiary dentin formation. rhEREG enhanced the odontoblastic differentiation of DPSCs following upregulated p38 MAPK and Erk1/2 phosphorylation, but not JNK, whereas depletion of EREG suppressed DPSC differentiation. Gefitinib decreased odontoblastic differentiation with decreased phosphorylation of p38 MAPK and Erk1/2. And suppression of p38 MAPK and Erk1/2 pathways attenuated DPSC differentiation. In human dental pulp tissue, EREG upregulation in deep caries correlates with odontoblastic differentiation enhancement.

**Conclusion:**

Epiregulin is released during tertiary dentin formation. And EREG enhanced DPSC odontoblastic differentiation via MAPK pathways.

## 1 INTRODUCTION

Dental caries is one of the most common diseases in dentistry,^1^, ^2^ which contributes to non‐vital teeth and further teeth missing. Indirect pulp capping (IPC), a vital pulp therapy (VPT), is always performed in the clinic to preserve pulp vitality and subsequent natural teeth. It is accomplished by applying pulp capping agents, usually calcium hydroxide, over the deep cavities to induce tertiary dentin formation in the underlying dental pulp, and ultimately protect pulp tissue from injurious stimuli from outside. This procedure relies on the odontogenic differentiation potential of dental pulp stem cells (DPSCs) in the pulp tissue.^3^ The insults, including trauma, chemicals and microbes, could stimulate the DPSCs to differentiate into odontoblasts, which secrete dentin matrix and form tertiary dentin eventually.^4^, ^5^ However, regardless of which materials are used in IPC, the production of tertiary dentin is a reaction of DPSCs to the high pH, unlike the natural dentinogenesis process. Furthermore, IPC is not always predictable, often resulting in the need for eventual endodontic therapy.^6^, ^7^ Consequently, the development of an improved stem cell‐based therapy that simulates the natural odontogenesis is expected.

The dentinogenesis is a delicate and dynamic process involving a number of precisely coordinated growth factors. Recent progress in understanding growth factors and signal molecules changes during stem cell‐based dentinogenesis and how they are imitated during tertiary dentin formation provide us insight of new strategies, exploiting growth factors as bioactive agents in VPT. With the identification of growth factors participating in fate choice of mesenchymal stem cells (MSCs), such as proliferation and differentiation, the epidermal growth factor (EGF) family is considered to be essential regulatory factor.^8^, ^9^ The EGF family, including EGF, transforming growth factor‐α (TGF‐α), epiregulin (EREG) and amphiregulin, regulates proliferation and cytodifferentiation via binding to epidermal growth factor receptor (EGFR) on cell membrane. This induces protein‐tyrosine kinase activity of EREG intracellular tyrosine residues and then stimulates the mitogen‐activated protein kinase (MAPK) signalling pathway.^10^, ^11^ In turn, the MAPK signalling initiates series of signal transduction cascades that give rise to various biological processes, such as MSCs proliferation/differentiation, tissue remodelling and wound repair.^8^, ^12^, ^13^, ^14^ Moreover, EGF increased extracellular matrix mineralization of DPSCs *in vitro*, indicating the significant role of EGF family in promoting differentiation of DPSCs.^15^

Epiregulin, a broad specificity EGF family member, has been shown as a more potent and more effective activator of MAPK signalling pathway than EGF or TGF‐α, especially for activating protein kinase B (Akt) and extracellular signal‐regulated kinase (Erk) in various biological processes.^16^ EREG has been indicated to be released by smooth muscle cells, activates Erk and p38 MAPK signalling pathways and acts as a critical regulator for dedifferentiation.^17^ Recent studies have found that EREG promoted osteogenic or dentinogenic differentiation and proliferation of human dental stem cells from the apical papilla (SCAP) through activating c‐Jun N‐terminal kinase (JNK) and mitogen‐activated protein kinase‐ERK kinase (MEK)/Erk.^18^, ^19^ Besides, our previous study compared gene expressions of fetal dental papilla cells (FDPCs) and human adult dental pulp cells (ADPCs) by human growth factor array, in which we found that the expression of *EREG* in FDPCs was significantly higher than that in ADPCs, and ADPCs complemented with EREG‐stimulated non‐dental epithelial cells differentiate to dental epithelial cells.^20^ These evidences indicated that EREG could be a promising bioactive molecule for dental tissue repair and regeneration. However, the effect of EREG on tertiary dentin formation and DPSC odontoblastic differentiation remains unknown.

Here, the present study was designed to detect the change in EREG during tertiary dentin formation using rat deep cavity model and explore the effect of EREG on DPSC odontoblastic differentiation. The potential role of MAPK signalling pathway in EREG‐enhanced odontoblastic differentiation was also analysed.

## 2 MATERIALS AND METHODS

### 2.1 Ethics statement

All experiments using animals followed guidelines approved by State Key Laboratory of Oral Diseases. All human tissues were collected at West China Hospital of Stomatology in accordance with the guidelines set by Sichuan University. The study was approved by the ethical committees of the West China School of Stomatology, Sichuan University and State Key Laboratory of Oral Diseases.

### 2.2 Deep cavity model with rat

The operative procedures were as described by D'Souza RN^21^ and modified. Adult male Sprague‐Dawley rats weighing from 150 to 220 g were used for this experiment. Animals were anesthetized with 10% chloral hydrate in double‐distilled H_2_O injected intraperitoneally at a dose of 50 mg/kg. Then, round cavities were prepared with a round bur (head diameter, 1.0 mm) on occlusal mesial surfaces of the maxillary and mandibular first molars, approximately half of the dentin thickness and remaining unrestored during the experiment period. One molar in each animal was left untreated, serving as a control. The rats were sacrificed 30 days after surgery.

### 2.3 Histological and immunohistochemical analysis

The rat first molar areas were dissected and fixed in 4% paraformaldehyde overnight at 4°C. Decalcified samples were processed and embedded in paraffin, and 5‐μm‐thick sections were prepared (model HM 340E; Microm) and stained with haematoxylin/eosin. Immunohistochemical staining was performed using HRP‐DAB Cell & Tissue Staining Kit (R&D Systems) according to the manufacturer's instructions. EREG expression was detected with rabbit anti‐EREG as the primary antibody (1:50; Abcam) and completed with a 5‐minutes incubation with 3,3′‐diaminobenzidine (DAB).

### 2.4 Enzyme‐linked immunosorbent assay

Fresh serum was collected from rats, and dental pulp tissue was harvested from rat first molars. Enzyme‐linked immunosorbent assay kit was purchased for EREG assay and was performed according to the manufacturer's protocols (Cloud‐Clone Corp).

### 2.5 Cell culture

Human third molars were collected at West China Hospital of Stomatology from patients for tooth extraction (n = 30, 13‐25 years of age), with informed consent. Human DPSCs were isolated and cultured based on the method previously reported.^3^ Briefly, dental pulp tissue was retrieved from the dental pulp cavity and digested with 3 mg/mL collagenase type I (Gibco) for 60 minutes at 37°C. Cells were maintained in Dulbecco's modified Eagle's medium (DMEM; Hyclone) containing 10% foetal bovine serum (FBS; Gibco) and antibiotics (100 U/mL penicillin–streptomycin; Hyclone) at 37°C in a 5% CO_2_ humidified atmosphere (negative control medium, NC). The odontogenic medium (OM) was DMEM supplemented with 10% FBS, 100 U/mL penicillin–streptomycin (Hyclone), 10 mmol/L sodium β‐glycerophosphate (Sigma‐Aldrich), 10 nmol/L dexamethasone (Sigma‐Aldrich) and 50 μg/mL l‐ascorbic acid (Sigma‐Aldrich). Recombinant human EREG protein (rhEREG) and gefitinib were purchased from R&D Systems and Selleck, respectively. Short hairpin RNAs (shRNA) of EREG were obtained from GeneCopoeia Inc Scramble shRNA was used as a blank control. The target sequences for shRNA were shEREG, 5′‐GGCTTTGACCGTGATTCTTAT‐3′; and scramble, 5′‐ACAGAAGCGATTGTTGATC‐3′. The signalling pathway‐specific inhibitors used in this study were p38 MAPK (SB203580; Selleck), JNK inhibitor (SP600125; Selleck) and Erk1/2 inhibitor (U0126; Selleck).

### 2.6 Alkaline phosphatase and alizarin red S staining

Dental pulp stem cells were cultured in OM for 14 days with or without rhEREG. For alkaline phosphatase (ALP) staining, cells were cultured for 7 days, fixed in 4% paraformaldehyde and stained with an ALP staining kit according to the manufacturer's protocol (Beyotime). ALP activity was quantified following the manufacturer's instructions (Beyotime). The total protein content was measured by BCA protein assay kit (Beyotime). ALP activity was normalized to the corresponding total protein content. The formation of mineralized nodules of cells cultured for 14 days was evaluated by alizarin red S staining (Cyagen). For quantitative analysis, 10% cetylpyridinium chloride (in 10 mmol/L sodium phosphate, pH 7.0) was added to destrain the alizarin red‐positive deposition. The absorbance was measured at OD = 562 nm.

### 2.7 Real‐time reverse transcription polymerase chain reaction analysis

Total RNAs were purified from DPSCs using TRIzol reagents (Invitrogen) and reverse‐transcribed to complementary DNA (cDNA) using the SuperScript first‐strand synthesis system (Life Technology). Real‐time reverse transcription polymerase chain reaction (real‐time RT‐PCR) was performed with Power SYBR Green PCR Master Mix (Takara Bio Inc) and an ABI 7900 system (Applied Biosystems), according to the manufacturer's instructions. All reactions were run in triplicate and normalized to glyceraldehyde 3‐phosphate dehydrogenase (GAPDH). Relative gene expression levels were calculated using ΔΔCt values. The primers for *EREG*, dentin sialophosphoprotein (*DSPP*), osteocalcin (*OCN*), runt‐related transcription factor 2 (*RUNX2*), osterix (*OSX*) and *GAPDH* were as follows: *EREG* (F: 5′‐ATCACAGTCGTCGGTTCCAC‐3′ and R: 5′‐AGGCACACTGTTATCCCTGC‐3′); *DSPP* (F: 5′‐ATATTGAGGGCTGGAATGGGGA‐3′ and R: 5′‐TTTGTGGCTCCAGCATTGTCA‐3′); *OCN* (F: 5′‐ATTGTGGCTCACCCTCCATC‐3′ and R: 5′‐CCAGCCTCCAGCACTGTTTA‐3′); *RUNX2* (F: 5′‐CCTTTACTTACACCCCGCCA‐3′ and R: 5′‐GGATCCTGACGAAGTGCCAT‐3′); *OSX* (F: 5′‐TCTGCGGGACTCAACAACTC‐3′ and R: 5′‐TAGCATAGCCTGAGGTGGGT‐3′); and *GAPDH* (F: 5′‐GGAGCGAGATCCCTCCAAAAT‐3′ and R: 5′‐GGCTGTTGTCATACTTCTCATGG‐3′).

### 2.8 Western blot analysis

Cells were lysed in RIPA buffer (50 mmol/L Tris [pH 8], 250 mmol/L NaCl, 0.05% sodium dodecyl sulphate, 0.5% deoxycholate, 1% NP‐40). Proteins were separated on a 10% Bis‐Tris protein gel (1.0 mm) and transferred to nitrocellulose membrane (Bio‐Rad). Antibodies used were p‐p38 MAPK (4631; CST), p38 MAPK (8690; CST), p‐JNK (4668; CST), JNK (9252; CST), p‐Erk1/2 (4370; CST), Erk1/2 (4695; CST) and α‐tubulin (41517; SAB). Horseradish peroxidase (HRP)‐conjugated secondary antibodies were purchased from ZSGB‐BIO. The membranes were detected with a chemiluminescent reagent kit. α‐Tubulin served as an internal control. For image analysis, the films were scanned with a GS‐700 imaging densitometer (Bio‐Rad). Relative protein expression levels were measured using ImageJ software (National Institute of Health).

### 2.9 Statistical analysis

All results are presented as means ± standard deviation (SD) of triplicate measurements. All experiments were repeated at least three times. The data were evaluated statistically using Student's *t* tests or one‐way ANOVA with the Tukey post hoc test.

## 3 RESULTS

### 3.1 EREG is activated during tertiary dentin formation

**Figure 1:**
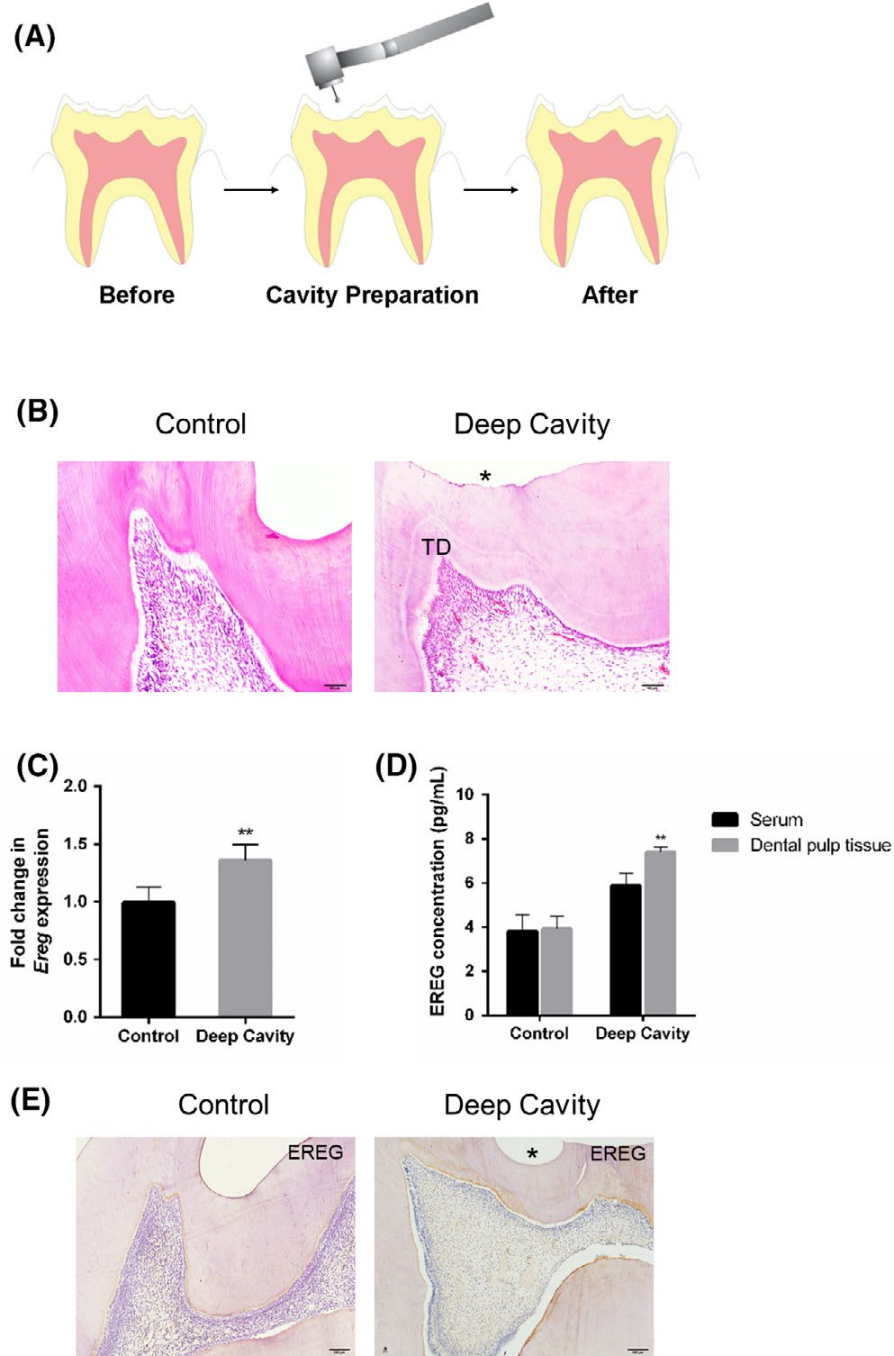
Epiregulin (EREG) is activated during tertiary dentin formation. A, Schematic diagrams indicating the experimental procedures for deep cavity preparation. B, Histological analysis of tertiary dentin formation at the cavity‐prepared area after 30 d by haematoxylin/eosin staining (n = 8). The asterisk (*) represents the cavity. TD, tertiary dentin. Scale bars = 50 μm. C, The expression level of *Ereg* in rat dental pulp tissue by real‐time RT‐PCR on 30 d post‐operation (n = 5). **P < .01. D, Ereg concentration in rat serum and rat dental pulp tissue was analysed using ELISA on 30 d post‐operation (n = 5). E, Ereg expression in rat dental pulp tissue by immunohistochemistry on 30 d post‐operation (n = 8). The asterisk (*) represents the cavity. Scale bars = 100 μm

To investigate tertiary dentin formation *in vivo*, the deep cavity model was established with rat first molars (Figure 1A). Adult male rats were divided into two groups: no deep cavity preparation (control group) and deep cavity preparation (experiment group). In the deep cavity group, tertiary dentin was observed beneath the dentin at deep cavity sites at 30 days post‐operation, the dentinal tubules lined up in disorder and irregularity; cells arranged irregularly in the mesial pulp horn, and capillary vessels under the cell layer expanded. On the contrary, newly formed mineralization tissue was rarely detected in the control group (Figure 1B).

The expression level of *Ereg* during tertiary dentin formation was detected with real‐time RT‐PCR and immunohistochemistry. The expression level of *Ereg* was remarkably increased in the deep cavity group compared with controls (Figure 1C). To further investigate the concentration of EREG released during this process, EREG secretion from dental pulp tissue and serum was quantified by ELISA (Figure 1D). The dental pulp with tertiary dentin formation released significantly more Ereg than control group (Figure 1E).

### 3.2 EREG enhances odontoblastic differentiation potential of DPSCs

**Figure 2:**
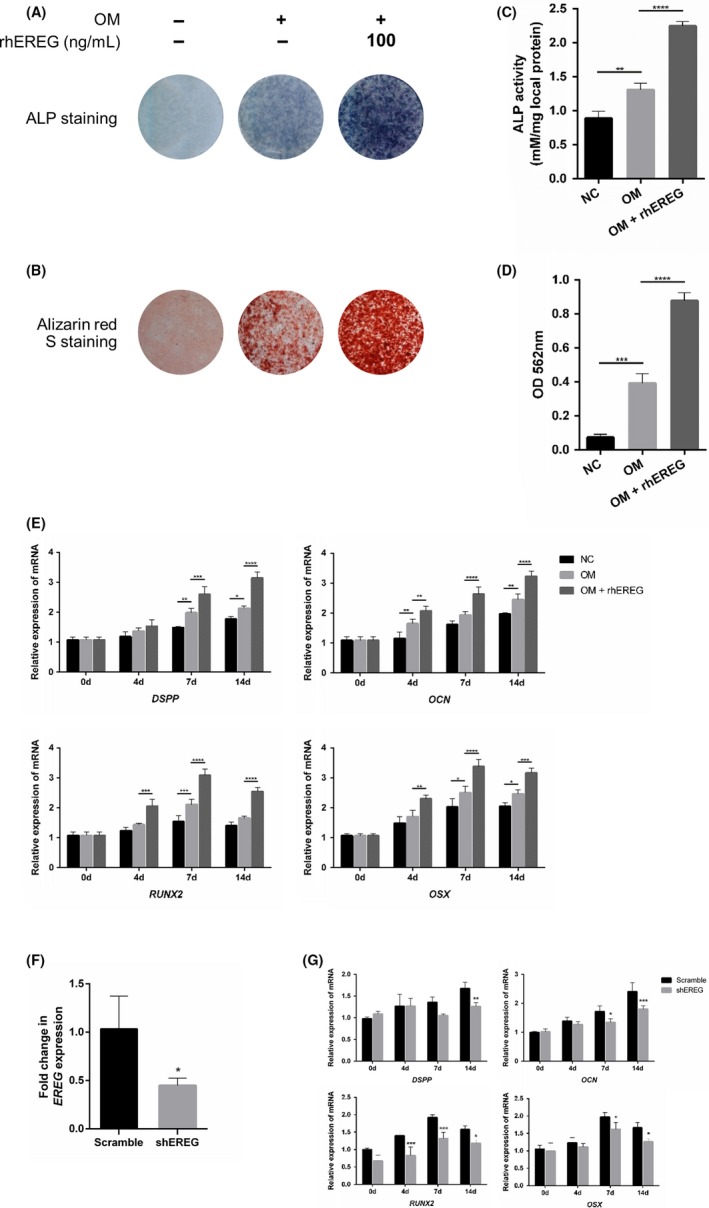
Epiregulin (EREG) enhances odontoblastic differentiation potential of dental pulp stem cells (DPSCs). A, alkaline phosphatase (ALP) staining on 7 d, and B, alizarin red S staining on 14 d of DPSCs cultured in NC, OM or OM with 100 ng/mL rhEREG (n = 3). C, The quantitative analysis of ALP activity (n = 3). D, The quantitative analysis of alizarin red S staining (n = 3). E, Expression levels of odontoblastic differentiation‐related markers, *DSPP*, *OCN*, *RUNX2, OSX*, were measured by real‐time RT‐PCR on 0, 4, 7 and 14 d of each culture condition (n = 3). F, Short hairpin RNAs were used to infect DPSCs in order to knockdown EREG (shEREG) or scramble shRNA (scramble). The knockdown efficiency of *EREG* gene expression in DPSCs, detected by real‐time RT‐PCR (n = 3). G, The changes in *DSPP*, *OCN*, *RUNX2* and *OSX* expression levels after the depletion of *EREG* were measured by real‐time RT‐PCR (n = 3). *DSPP*, dentin sialophosphoprotein; NC, negative control medium; *OCN*, osteocalcin; OM, odontogenic medium; *OSX*, osterix; rhEREG, recombinant human EREG protein; *RUNX2*, runt‐related transcription factor 2. The data are presented as means ± SD. *P < .05, **P < .01, ***P < .001, ****P < .0001

Dental pulp stem cells were collected from human dental pulp tissue and cultured in OM for 2 weeks with or without rhEREG (0, 25, 50, 100, 150 or 200 ng/mL). The ALP staining in response to administration of EREG was conducted on day 7. Compared with NC, OM and OM with 25, 50 ng/mL rhEREG groups, ALP staining revealed that ALP activity was significantly higher with administration of 100, 150 or 200 ng/mL rhEREG. And there were no significant differences presented among 100, 150 and 200 ng/mL rhEREG groups (Figure S1A). The quantitative analysis with ALP activity assay confirmed the results of staining (Figure S1C). Alizarin red S staining and quantitative results also showed that the groups with 100, 150 and 200 ng/mL rhEREG treatments for 14 days presented more mineralized nodules, compared with NC, OM and OM with 25, 50 ng/mL rhEREG groups. Similarly, there were no significant differences shown among 100, 150 and 200 ng/mL rhEREG groups (Figure S1B, D). In addition, real‐time RT‐PCR was performed on days 0, 4, 7 and 14 to detect the mRNA expression of odontoblastic differentiation markers, including *DSPP*, *OCN*, *RUNX2* and *OSX*. Consistent with ALP activity and alizarin red S staining, administration of 100, 150 and 200 ng/mL rhEREG significantly increased the expression levels of these genes in DPSCs compared with other groups (Figure S1E). Since 100, 150 or 200 ng/mL rhEREG presented equivalent effects on odontoblastic differentiation of DPSCs, we selected 100 ng/mL rhEREG in our further study (Figure 2A‐E).

To further confirm the efforts of EREG during odontoblastic differentiation in DPSCs, we knocked down EREG in DPSCs with lentiviral EREG short hairpin RNAs (shEREG). The knockdown efficiency of *EREG* in DPSCs was validated by real‐time RT‐PCR (Figure 2F). And EREG depletion caused reductions in *DSPP*, *OCN*, *RUNX2* and *OSX* mRNA expression in DPSCs (Figure 2G).

### 3.3 The EGFR inhibitor gefitinib suppresses EREG‐mediated odontoblastic differentiation enhancement of DPSCs

**Figure 3:**
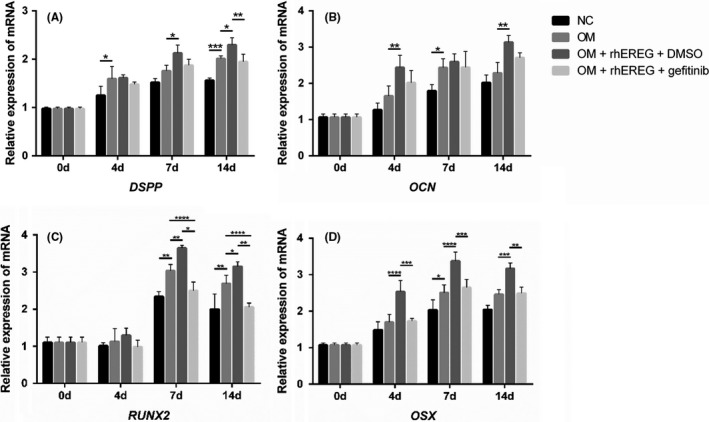
The Epiregulin (EREG) inhibitor gefitinib suppresses EREG‐mediated odontoblastic differentiation enhancement of dental pulp stem cells (DPSCs). Gefitinib was used to inhibit the EREG receptor EGFR. DPSCs were treated with gefitinib (2 μmol/L) for 48 h and then cultured with 100 ng/mL rhEREG for 14 d (n = 3). Real‐time RT‐PCR was performed for the expression levels of *DSPP*, *OCN*, *RUNX2* and *OSX* (n = 3). *DSPP*, dentin sialophosphoprotein; *OCN*, osteocalcin; *OSX*, osterix; rhEREG, recombinant human EREG protein; *RUNX2*, runt‐related transcription factor 2. The data are presented as means ± SD. *P < .05, **P < .01, ***P < .001, ****P < .0001

As EREG is known to activate EGFR as its ligand, we used EGFR‐tyrosine kinase inhibitor gefitinib to examine whether EREG could activate EGFR and promote subsequent odontoblastic differentiation. DPSCs were cultured in OM supplemented with 100 ng/mL rhEREG with or without gefitinib. Real‐time RT‐PCR was also performed on days 0, 4, 7 and 14 to examine the mRNA expression of odontoblastic differentiation markers as described above. Results of real‐time RT‐PCR showed that gefitinib treatment suppressed EREG‐enhanced *DSPP*, *RUNX2* and *OSX* expression (Figure 3A, C, D). However, no difference was observed in the expression of *OCN* (Figure 3B). These results confirmed that the differentiation enhancement was suppressed with the EGFR inhibitor gefitinib treatment.

### 3.4 EREG enhances DPSC odontoblastic differentiation in an MAPK‐dependent manner

**Figure 4:**
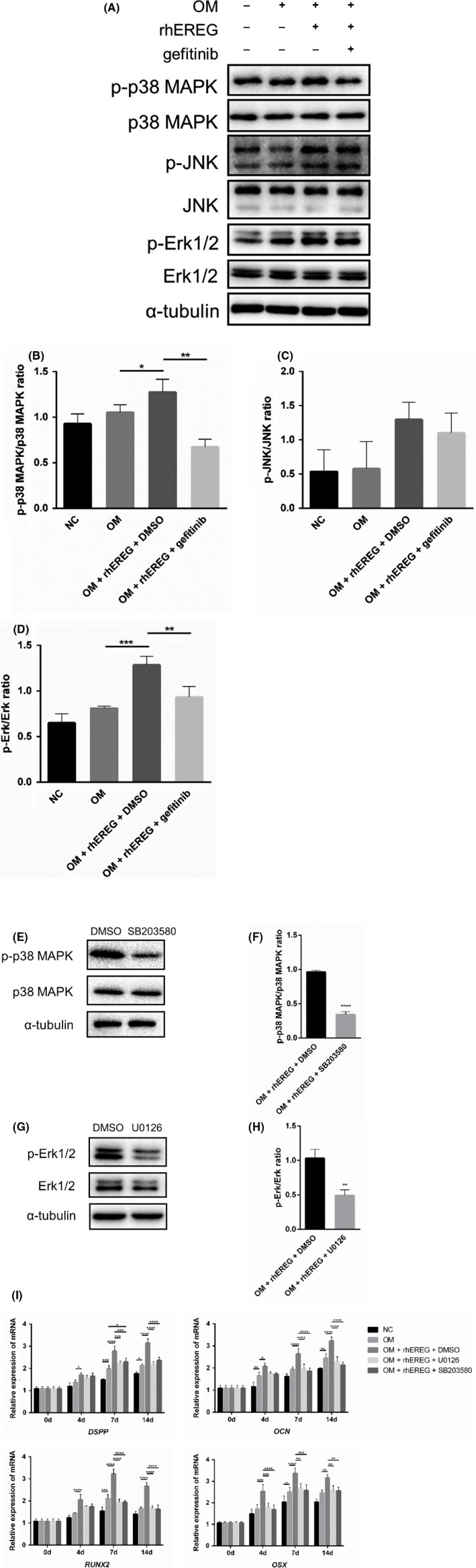
Epiregulin (EREG) enhances dental pulp stem cells (DPSCs) odontoblastic differentiation in an MAPK‐dependent manner. A, Western blot analysis of p‐p38 MAPK, p38 MAPK, p‐JNK, JNK, p‐Erk1/2 and Erk1/2 from DPSCs pre‐treated with specific inhibitors separately for 1 h followed by stimulation with rhEREG for 2 h. α‐Tubulin served as an internal control. B, C, D, The quantitative protein analysis was performed for the Western blot. E, G, Western blot analysis of p‐p38 MAPK (E), and p‐Erk1/2 (G) in DPSCs after being treated with p38 MAPK signalling pathway inhibitor SB203580, and Erk1/2 signalling pathway inhibitor U0126 (20μM) for 1 h, respectively. α‐Tubulin served as an internal control. F, H, The quantitative protein analysis was performed for the Western blot. I, Real‐time RT‐PCR analysis of *DSPP*, *OCN*, *RUNX2* and *OSX* expression levels in DPSCs after being treated with p38 MAPK inhibitor SB203580, and Erk1/2 inhibitor U0126 (20 μmol/L) for 48h, and then cultured with 100 ng/mL rhEREG for 14 d (n = 3). p38 MAPK, p38 mitogen‐activated protein kinase; JNK, c‐Jun N‐terminal kinase; Erk1/2, extracellular signal‐regulated kinase 1/2. *DSPP*, dentin sialophosphoprotein; *OCN*, osteocalcin; *OSX*, osterix; rhEREG, recombinant human EREG protein; *RUNX2*, runt‐related transcription factor 2. The data are presented as means ± SD. *P < .05, **P < .01, ***P < .001, ****P < .0001

Then, we explored the mechanism of EREG‐enhanced odontoblastic differentiation potential of DPSCs. DPSCs were treated with 100 ng/mL of rhEREG. Western blotting was carried out to examine the protein expression levels of critical members of MAPK pathway, which showed that rhEREG enhanced phosphorylation of p38 MAPK and Erk1/2, since the levels of phosphorylated p38 MAPK and Erk1/2 were upregulated. However, no obvious changes were observed in phosphorylated JNK and total amount of p38 MAPK, JNK and Erk1/2 protein. Additionally, gefitinib suppressed the phosphorylation of p38 MAPK and Erk1/2, but expression level of phosphorylated JNK and expression of total p38 MAPK, JNK and Erk1/2 were not affected (Figure 4A‐D).

To further validate our findings, p38 MAPK or Erk1/2 was inhibited with specific inhibitor SB203580 or U0126, respectively. DPSCs were pre‐treated with 20 µmol/L inhibitors for 1 hour separately, and then, 100 ng/mL rhEREG was added. Western blot analysis indicated that these specific inhibitors blocked corresponding target efficiently (Figure 4E‐H). Real‐time RT‐PCR was conducted to investigate the effects on odontoblastic differentiation of DPSCs following p38 MAPK or Erk1/2 inhibitor treatments. Real‐time RT‐PCR showed that p38 MAPK inhibitor SB203580 dramatically suppressed EREG‐mediated enhancement of *DSPP*, *OCN*, *RUNX2* and *OSX* expression in DPSCs (Figure 4I). Similarly, Erk1/2‐specific inhibitor U0126 suppressed EREG‐enhanced the expression of *DSPP*, *OCN*, *RUNX2* and *OSX* in DPSCs (Figure 4I). These results indicated that EREG‐enhanced odontoblastic differentiation of DPSCs was repressed by p38 MAPK or Erk1/2 inhibition.

### 3.5 *EREG* upregulation correlates with enhanced odontogenesis in human dental pulp tissue

**Figure 5:**
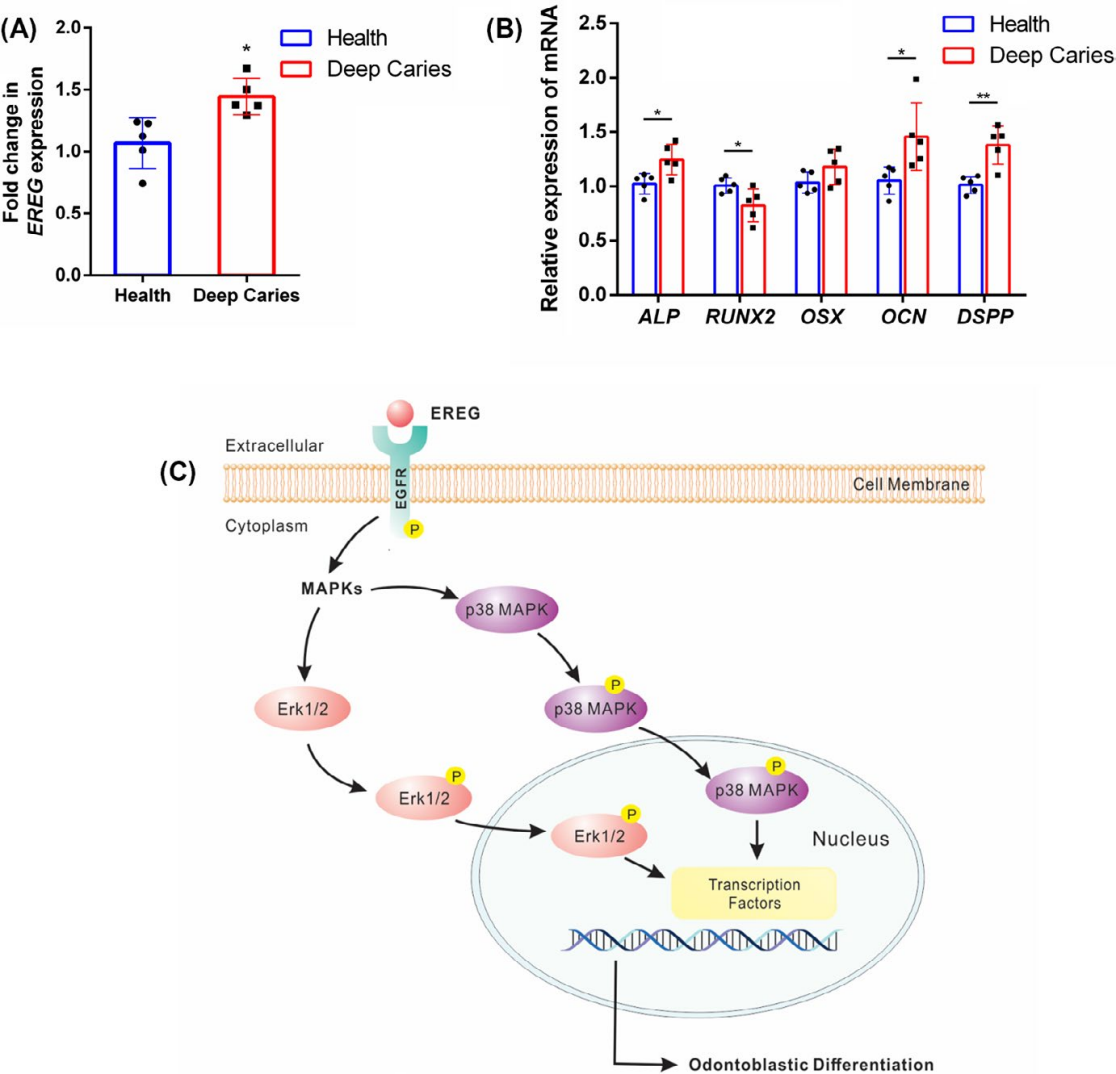
*EREG* upregulation correlates with enhanced odontogenesis in human dental pulp tissue. A, *EREG* expression in human dental pulp tissue from teeth with deep caries and healthy teeth by real‐time RT‐PCR (n = 5). B, Real‐time RT‐PCR was performed for the expression levels of *DSPP*, *OCN*, *RUNX2* and *OSX* in human dental pulp tissue from teeth with deep caries and healthy teeth (n = 5). The data are presented as means ± SD. *P < .05, **P < .01. C, Schematic illustration of the molecular mechanism of EREG on odontoblastic differentiation of dental pulp stem cells. *DSPP*, dentin sialophosphoprotein; *OCN*, osteocalcin; *OSX*, osterix; rhEREG, recombinant human EREG protein; *RUNX2*, runt‐related transcription factor 2

We collected human dental pulp tissue from teeth with deep caries (n = 5) and healthy teeth (n = 5), respectively. *EREG* expression level was detected by real‐time RT‐PCR in two groups. On average with five samples tested, a significantly higher expression of EREG was observed in dental pulp tissue from the deep caries group (Figure 5A). What is more, the higher level of *EREG* expression in deep caries group was correlated with the upregulation of odontoblastic differentiation markers, including *ALP*, *OCN* and *DSPP* (Figure 5B). *RUNX2* expression was reduced in deep caries group, and no significant change was observed in *OSX* expression between the health group and deep caries group (Figure 5B).

## 4 DISCUSSION

The aims of VPT are to preserve the dental pulp vitality, to induce DPSCs to differentiate into odontoblasts and ultimately form the tertiary dentin.^22^ Currently, VPT, including direct pulp capping, apexogenesis and IPC, depends on the multipotential differentiation of stem cells. However, unlike the natural dentinogenesis, dental pulp tissue responses to the high pH in IPC, which is unpredictable, and therefore resulting in the need for eventual endodontic therapy.^6^, ^7^ With the understanding of growth factors regulating dentinogenesis during tooth development, the application of growth factors offers opportunities for the development of novel strategies in VPT. To reveal the potential growth factors involving odontogenesis, the previous study of our group profiled the expression levels of growth factors in FDPCs and ADPCs, dental mesenchymal cell populations from different cytodifferentiation stages of dentinogenesis, and indicated the expression of *EREG* was significantly higher in FDPCs compared with ADPCs.^20^ Furthermore, we demonstrated that EREG re‐established the inductive potential of ADPCs, suggesting that EREG is involved in regulating dental mesenchymal cells during odontogenesis. In addition, EREG has been indicated to promote SCAP proliferation.^18^ Therefore, we assumed that EREG might be a crucial regulatory factor of DPSC differentiation. To identify whether EREG is responsible for triggering this stimulation of odontoblastic differentiation, we established the deep cavity model with rat first molar, in which the tertiary dentin was observed. With this animal model, we found that large quantities of EREG were released during tertiary dentin formation. Besides, gain‐ and loss‐of‐function studies indicated that rhEREG upregulated the expression levels of odontoblastic differentiation markers of DPSCs, including *DSPP*, *OCN*, *RUNX2* and *OSX*, and suggested the odontoblastic differentiation potential of EREG in DPSCs.

Epiregulin is a member of EGF family, and always binding with EGFR to regulate various biological processes, including cell growth, motility, proliferation and differentiation.^23^, ^24^, ^25^ Here, we blocked EGFR with a specific inhibitor, gefitinib, suppressed EREG‐enhanced odontoblastic differentiation of DPSCs, which confirmed that EREG transduced its signalling and apply its effect by binding to EGFR. The previous study has indicated that EREG could activate MEK/Erk and JNK signalling pathways, consequently stimulating SCAP proliferation and osteogenic differentiation.^18^, ^19^ And EREG has been suggested to be of importance in inflammation, wound recovery and smooth muscle regeneration.^8^, ^12^, ^13^ It could be released by smooth muscle cells, activate Erk and p38 MAPK signalling, and play a pivotal role in dedifferentiation.^17^ To determine whether MAPK signalling pathway was involved in EREG‐enhanced odontoblastic differentiation of DPSCs, we detected the changes in three major subfamilies, p38 MAPK, JNK and Erk1/2, during odontoblastic differentiation. Our results showed that rhEREG activated MAPK signalling pathway by upregulating phosphorylation of p38 MAPK and Erk1/2, and knockdown of EREG suppressed the expression of phosphorylated p38 MAPK and Erk1/2, but not JNK. Moreover, suppression of p38 MAPK or Erk1/2 attenuated the influence of rhEREG on DPSC odontoblastic differentiation, considering the decreased expression levels of *DSPP, OCN, RUNX2* and *OSX*, thereby providing further evidence that MAPK signalling pathway plays a vital role in EREG‐enhanced odontoblastic differentiation of DPSCs. Above all, these results indicated that EREG‐enhanced odontoblastic differentiation of DPSCs depends on stimulation of p38 MAPK and Erk1/2, not JNK signalling pathway (Figure 5C).

Cell differentiation is a complex process involving coordinating network of growth factors and signalling pathways. MAPK signalling involving a set of serine/threonine kinases plays a crucial part in cytodifferentiation, by transmitting various extracellular stimuli signals from the cell membrane into the nucleus.^26^ Erk1/2 signalling pathway represents one of the most characteristic MAPK signalling pathways and has been demonstrated to be involved in the regulation of cytodifferentiation. Generally, MAPKs are activated through motivation of tyrosine kinase receptors (RTKs) in a multistep process. Erk1/2 is phosphorylated by MEKs, the essential linkers for EGF to MAP kinase, sequentially increasing Erk activity. Then, activated Erk enters cell nucleus and activates transcription of related gene regulating cell proliferation, differentiation and mitosis.^27^ Brain‐derived neurotrophic factor motivates human umbilical cord blood‐derived MSCs differentiation through Erk‐dependent signalling pathway.^28^ And Erk1/2 signalling pathway has been indicated as a key regulator in osteogenic differentiation of periodontal ligament stem cells (PDLSCs).^29^ Relevant studies also revealed that Erk1/2 signalling pathway participates in the regulation of cell differentiation, such as the VEGF‐A/VEGFR2‐enhanced differentiation of adipose‐derived MSCs^30^ and the mineral trioxide aggregate‐upregulated odonto/osteogenic capacity of bone marrow MSCs (BMSCs).^31^ The other major MAPKs family, p38 MAPK, is considered as ‘stress‐activated signalling pathways’ (SAPKs), since it is always activated by various environmental stresses, including oxidative stress, UV irradiation, hypoxia and ischaemia, but to a lesser extent by growth factors.^32^ The previous studies have indicated that p38 MAPK signalling pathway is associated with anti‐proliferative and apoptotic functions,^33^ and has opposite effects to Erk1/2. Inhibition of p38 MAPK increased Erk1/2 phosphorylation, whereas Erk1/2 inhibition induced p38 MAPK phosphorylation and promoted osteogenic differentiation of BMSCs, which suggests a crosstalk between Erk and p38 signalling pathways.^34^ What is more, it was reported that p38 MAPK was involved in pro‐survival functions, such as promoting differentiation.^35^, ^36^ The activation of both Erk1/2 and p38 MAPK signalling is required during the myricetin‐mediated osteogenic differentiation enhancement of human PDLSCs.^37^ Besides, p38 MAPK signalling pathways play a greater role than Erk1/2 in hypoxic‐mediated BMSC osteogenic differentiation.^38^ In the present study, our results identified the critical role of p38 MAPK and Erk1/2 signalling pathways in EREG‐enhanced odontoblastic differentiation of DPSCs, but further in‐depth research is necessary to explore detailed mechanisms in this process.

The regeneration of dentin runs through the entire process of both physical and pathological changes in teeth. In order to protect the underlying dental pulp from external stimuli, such as deep caries, tertiary dentin formation by the odontoblastic differentiation of DPSCs is of great concern. In our experiment, we collected human dental pulp tissue from healthy teeth and teeth with deep caries. Odontoblastic differentiation markers were found upregulated in teeth with deep caries. However, unlike the *in vitro* results, the gene expression level of *RUNX2* was reduced with deep caries. RUNX2 is a known specific transcription factor that regulates the differentiation of MSCs, closely related to the differentiation of osteoblasts and odontoblasts.^39^, ^40^ Studies have found that *Runx2* knockout mice died after birth, and the tooth development stagnated at the immature phase of odontoblast differentiation.^41^ Besides, it has been reported that *RUNX2* expression showed obvious spatial and temporal specificity during the repair of dental pulp injuries, highly expressed at the early stages of odontoblastic differentiation, while significantly decreased at the late stage.^40^ Teeth with deep caries induce tertiary dentin formation in the underlying dental pulp tissue, which are at the late stage of odontoblastic differentiation. Human dental pulp tissue collected in this study was from teeth with deep caries clinically, which was supposed at the late stage of odontoblastic differentiation. These explain why the expression of *RUNX2* was lower in dental pulp tissue from teeth with deep caries compared with healthy teeth.

Taken together, our study revealed that EREG could promote the DPSC odontoblastic differentiation by stimulating MAPK signalling pathway, especially with p38 MAPK and Erk1/2 signalling. These results indicate that EREG is of great significance in promoting odontoblastic differentiation and provide new insights into the modifications of vital pulp therapy. Further *in vivo* studies should be performed with animal models to explore the effects of EREG on the tertiary dentin formation before future clinical applications.

## 5 CONCLUSIONS

In conclusion, the expression level of *EREG* was increased during the tertiary dentin formation. EREG enhances differentiation of DPSCs into odontoblasts via activating p38 MAPK and Erk1/2 signalling pathways.

## CONFLICTS OF INTEREST

The authors declare that they have no competing interests.

## AUTHOR CONTRIBUTIONS

Dixin Cui and Mian Wan conceived, designed, acquired, analysed, interpreted and drafted the data and critically revised the manuscript; Jiani Xiao acquired, analysed, interpreted and drafted the manuscript; Yachuan Zhou designed, acquired, analysed and drafted the manuscript; Xin Zhou analysed and interpreted the data and drafted the manuscript; Ying Liu acquired and analysed the data; Yiran Peng analysed the data and drafted the manuscript; Yi Yu and Hongyu Li acquired and analysed the data; Xuedong Zhou and Quan Yuan conceived and designed the data; Liwei Zheng conceived, designed and drafted the data and critically revised the manuscript.

## Data Availability

Raw data were generated at State Key Laboratory of Oral Diseases & National Clinical Research Center for Oral Diseases, West China Hospital of Stomatology, Sichuan University. Derived data supporting the findings of this study are available from the corresponding author, Liwei Zheng, on request.
