# Supplementary figures and images for: Epiregulin enhances odontoblastic differentiation of dental pulp stem cells via activating MAPK signalling pathway
Source: Cell Prolif. 2019 Aug 27;52(6):e12680. doi: 10.1111/cpr.12680 (PMC6869433; doi:10.1111/cpr.12680)

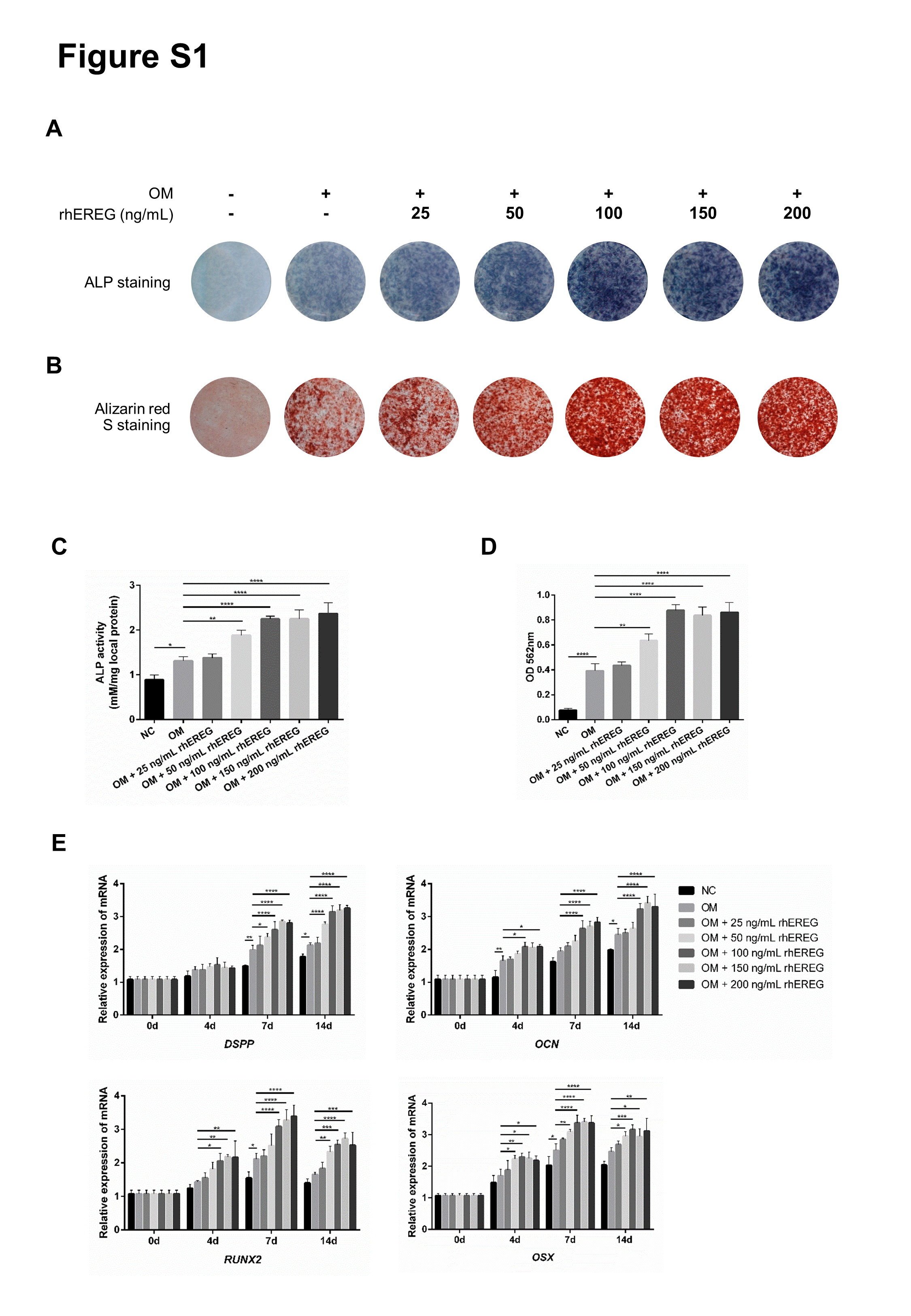

Supplement: Supplementary file 1 [file CPR-52-e12680-s001.tif]

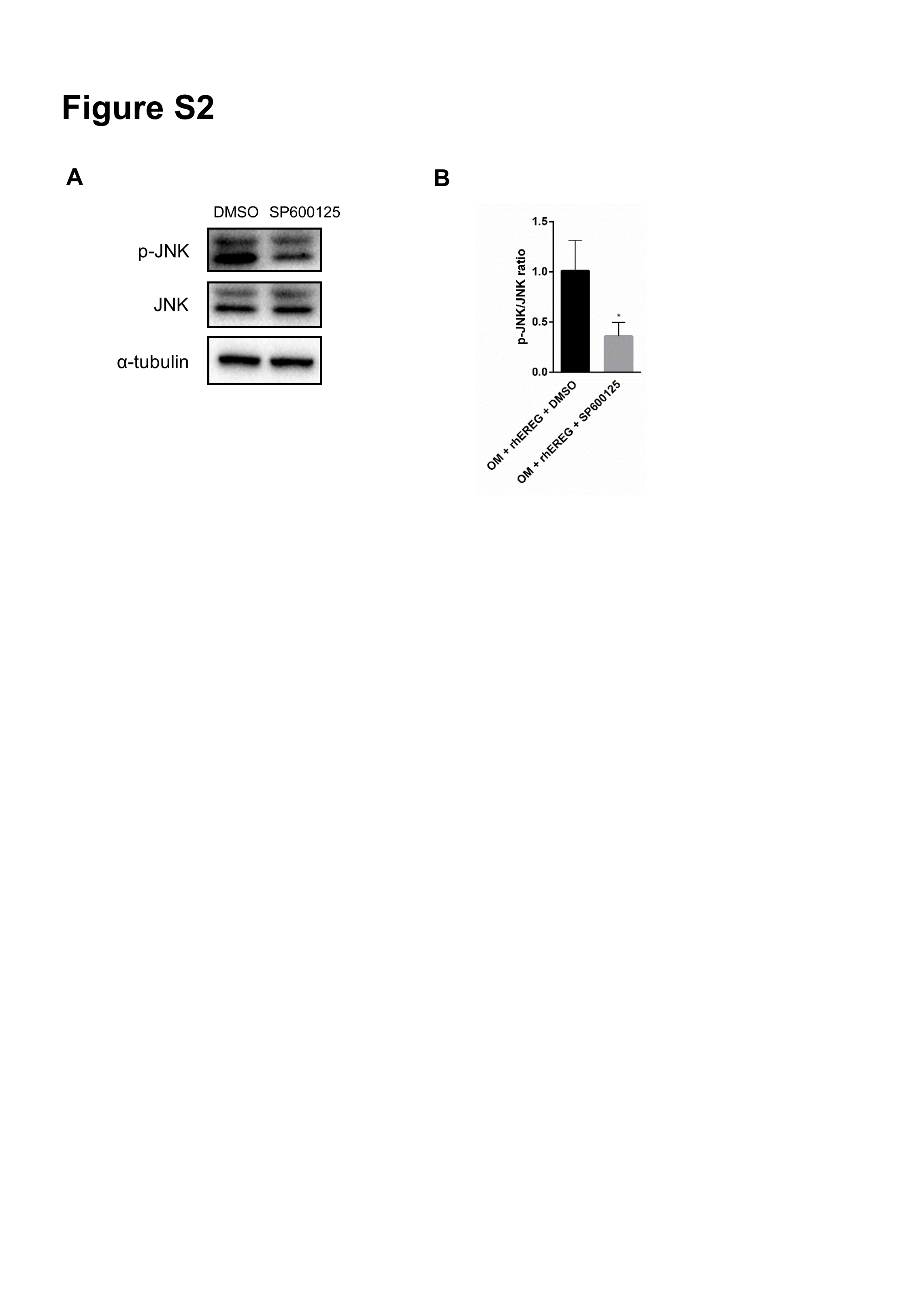

Supplement: Supplementary file 2 [file CPR-52-e12680-s002.tif]
